# Trajectories of functional decline in older adults with neuropsychiatric and cardiovascular multimorbidity: A Swedish cohort study

**DOI:** 10.1371/journal.pmed.1002503

**Published:** 2018-03-06

**Authors:** Davide L. Vetrano, Debora Rizzuto, Amaia Calderón-Larrañaga, Graziano Onder, Anna-Karin Welmer, Roberto Bernabei, Alessandra Marengoni, Laura Fratiglioni

**Affiliations:** 1 Aging Research Center, Department of Neurobiology, Care Sciences and Society, Karolinska Institutet and Stockholm University, Stockholm, Sweden; 2 Department of Geriatrics, Catholic University of Rome, Rome, Italy; 3 Division of Physiotherapy, Department of Neurobiology, Care Sciences and Society, Karolinska Institutet, Stockholm, Sweden; 4 Karolinska University Hospital, Stockholm, Sweden; 5 Stockholm Gerontology Research Center, Stockholm, Sweden; 6 Department of Clinical and Experimental Sciences, University of Brescia, Brescia, Italy; Scripps Translational Science Institute, UNITED STATES

## Abstract

**Background:**

Functional decline is a strong health determinant in older adults, and chronic diseases play a major role in this age-related phenomenon. In this study, we explored possible clinical pathways underlying functional heterogeneity in older adults by quantifying the impact of cardiovascular (CV) and neuropsychiatric (NP) chronic diseases and their co-occurrence on trajectories of functional decline.

**Methods and findings:**

We studied 2,385 people ≥60 years (range 60–101 years) participating in the Swedish National study of Aging and Care in Kungsholmen (SNAC-K). Participants underwent clinical examination at baseline (2001–2004) and every 3 or 6 years for up to 9 years. We grouped participants on the basis of 7 mutually exclusive clinical patterns of 0, 1, or more CV and NP diseases and their co-occurrence, from a group without any CV and NP disease to a group characterised by the presence of CV or NP multimorbidity, accompanied by at least 1 other CV or NP disorder. The group with no CV and/or NP diseases served as the reference group. Functional decline was estimated over 9 years of follow-up by measuring mobility (walking speed, m/s) and independence (ability to carry out six activities of daily living [ADL]). Mixed-effect linear regression models were used (1) to explore the individual-level prognostic predictivity of the different CV and NP clinical patterns at baseline and (2) to quantify the association between the clinical patterns and functional decline at the group level by entering the clinical patterns as time-varying measures. During the 9-year follow-up, participants with multiple CV and NP diseases had the steepest decline in walking speed (up to 0.7 m/s; *p* < 0.001) and ADL independence (up to three impairments in ADL, *p* < 0.001) (reference group: participants without any CV and NP disease). When the clinical patterns were analyzed as time varying, isolated CV multimorbidity impacted only walking speed (β −0.1; *p* < 0.001). Conversely, all the clinical patterns that included at least 1 NP disease were significantly associated with decline in both walking speed (β −0.21–−0.08; *p* < 0.001) and ADL independence (β −0.27–−0.06; *p* < 0.05). Groups with the most complex clinical patterns had 5%–20% lower functioning at follow-up than the reference group. Key limitations of the study include that we did not take into account the specific weight of single diseases and their severity and that the exclusion of participants with less than 2 assessments may have led to an underestimation of the tested associations.

**Conclusions:**

In older adults, different patterns of CV and NP morbidity lead to different trajectories of functional decline over time, a finding that explains part of the heterogeneity observed in older adults’ functionality. NP diseases, alone or in association, are prevalent and major determinants of functional decline, whereas isolated CV multimorbidity is associated only with declines in mobility.

## Abstract 2

Using data from a Swedish cohort study, Davide Vetrano and colleagues examine trajectories of functional decline in older adults with neuropsychiatric and cardiovascular multimorbidity.

## Author summary

**Why was this study done?:**

- The accumulation of chronic diseases, a condition called multimorbidity, is common in older age. Multimorbidity has a negative impact on older people’s health and quality of life.
- Cardiovascular and neuropsychiatric diseases are common and burdensome in older people and tend to cluster in the same persons.
- Understanding the associations between cardiovascular and neuropsychiatric multimorbidity and functional decline may help to detect groups of older people with poor prognosis in a timely fashion.

**What did the researchers do and find?:**

- We studied how the increasing number and the combination of cardiovascular and neuropsychiatric diseases are associated with two important measures of physical function in older people: walking speed and activities of daily living.
- We found that older people with 1 or more neuropsychiatric diseases experience a faster functional decline than those with cardiovascular diseases. However, when these two groups of diseases co-occurred, they played a synergistic role in speeding functional decline.

**What do these findings mean?:**

- Neuropsychiatric diseases are major predisposing factors for functional decline in older people. Both patients and physicians should be aware of that.
- The prompt identification of people with neuropsychiatric multimorbidity, especially in presence of cardiovascular diseases, may help to buffer the rapid functional decline they are likely to experience.

## Introduction

Aging is accompanied by a number of cellular and molecular dysfunctions that are responsible for a progressive loss of physical and cognitive resilience [1]. When death does not occur, physical decline and disability become progressively more frequent and strongly impact people’s health [2]. However, even if the direction of this process is certain, its pace varies among individuals, and both individual and contextual factors play a role in this variation [2]. The co-occurrence of multiple age-related chronic diseases is one of these factors.

Approximately 55%–98% of people aged ≥60 years have at least two chronic diseases (multimorbidity) [3,4]. Chronic diseases result from the interplay between people’s genetic susceptibility and lifelong environmental exposure [5]. The accumulation of chronic diseases over time is associated with progressive dysfunction in several systems and organs. Moreover, the co-occurrence of chronic diseases has negative effects on quality of life, frequency of healthcare use, dependency, and survival, which are greater than the sum of the effects of the individual diseases [1,4–6]. A large collaborative study that included 1.2 million participants reported that co-occurring cardio-metabolic diseases had a multiplicative negative impact on survival [7]. Indeed, chronic diseases do not appear at random but follow specific patterns due to shared risk factors and common pathophysiological pathways [4,8]. Previous research has consistently found that clusters of cardiovascular (CV) and neuropsychiatric (NP) disease constitute the major patterns of chronic disease in older adults [8].

CV and NP diseases are among the main contributors to the global burden of disability-adjusted life years (DALYs) in older adults. CV diseases are responsible for 30% and NP diseases for 7% of the years older people live with disability. Because of the swift growth of the older population, the overall contribution of these diseases to DALYs is expected to double by 2030 [9–10].

The impact of multimorbidity on functional ability has been investigated in several previous studies. With few exceptions [11–13], the majority reported that functional disability increased along with the number of chronic conditions [14–17]. However, few studies have investigated the relationship between multimorbidity and physical performance, and fewer have attempted to identify the specific clinical patterns associated with worse prognosis [2,18]. We intend to help fill this gap by identifying specific health profiles that lead to a major risk of functional decline.

The current study thus aimed to detect the effect and to quantify the impact of different clinical patterns—characterised by the co-occurrence of chronic CV and NP diseases—on trajectories of functional decline in older adults. Specifically, we studied the co-occurrence of CV and NP diseases across three different domains of increasing complexity: (1) pure CV morbidity, (2) pure NP morbidity, and (3) combined CV/NP multimorbidity. Studying the joint impact and prognosis of major chronic diseases will provide valuable information to aid clinicians in their daily counselling and help healthcare systems better target at-risk populations [8].

## Materials and methods

### Study design and population

**Fig 1:**
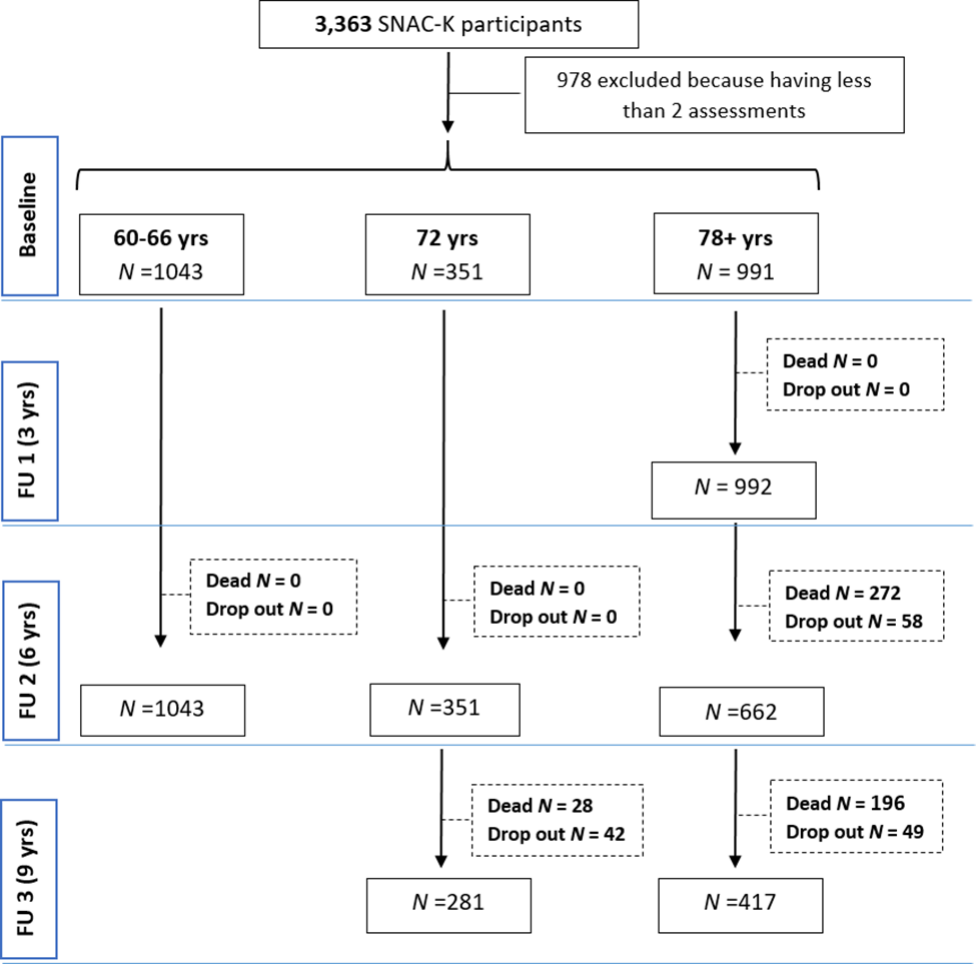
Flowchart of study participation over 9 years. Dropouts are due to either refusal of the participant/relative, loss of contact with the participant, or moving of the participant from the city where the study took place. FU1, follow-up 1; FU2, follow-up 2; FU3, follow-up 3; SNAC-K, Swedish National study of Aging and Care in Kungsholmen.

We used data from the 9-year follow-up of the population-based Swedish National study of Aging and Care in Kungsholmen (SNAC-K) [19]. This study consists of community-dwelling and institutionalised older adults ≥60 years. A random sample from 11 age cohorts born between 1892 and 1939 (the youngest and the oldest age cohorts were oversampled) living in the Kungsholmen district (Stockholm, Sweden) was invited to participate to the study. Those who accepted were evaluated for the first time between 2001 and 2004 and then followed up every 6 years (those aged <78 years) or every 3 years (those aged ≥78 years) [19]. At baseline, 3,363 people were examined (participation rate, 73%; **S1 Text**); 978 participants were excluded because they had been evaluated fewer than 2 times. Those excluded were older and more likely to be female, to live in an institution, and to have more chronic diseases than those included in the study (*p* < 0.001 for all). **Fig 1** depicts the flow of participants through the study. The study was approved by the Regional Ethics Review Board in Stockholm. Participants in the study completed a written informed consent form as stipulated in the ethical approval. For participants with prevalent or incident cognitive impairment, consent was obtained from the next of kin. [19]. The results of the present study were reported in accordance with the STROBE guidelines (**S1 Checklist**).

### Chronic disease assessment

During baseline and follow-up visits, SNAC-K participants underwent comprehensive clinical and functional assessments carried out by trained physicians, nurses, and neuropsychologists. Physicians collected information on diagnoses via physical examination, medical history, examination of medical charts, self-reported information, and/or proxy interviews [19]. Clinical parameters, lab tests, medications, and inpatient and outpatient care data from the Swedish National Patient Register were also used to identify specific conditions [20]. All diagnoses were coded in accordance with the International Classification of Diseases, 10th revision (ICD-10), and classified in accordance with a clinically driven methodology recently proposed by our group [20]. After this procedure, we identified 7 CV and 12 NP chronic diseases. Chronic conditions deemed to be risk factors were excluded (e.g., hypertension and dyslipidaemia; see **S2 Text** for details) [20].

**Fig 2:**
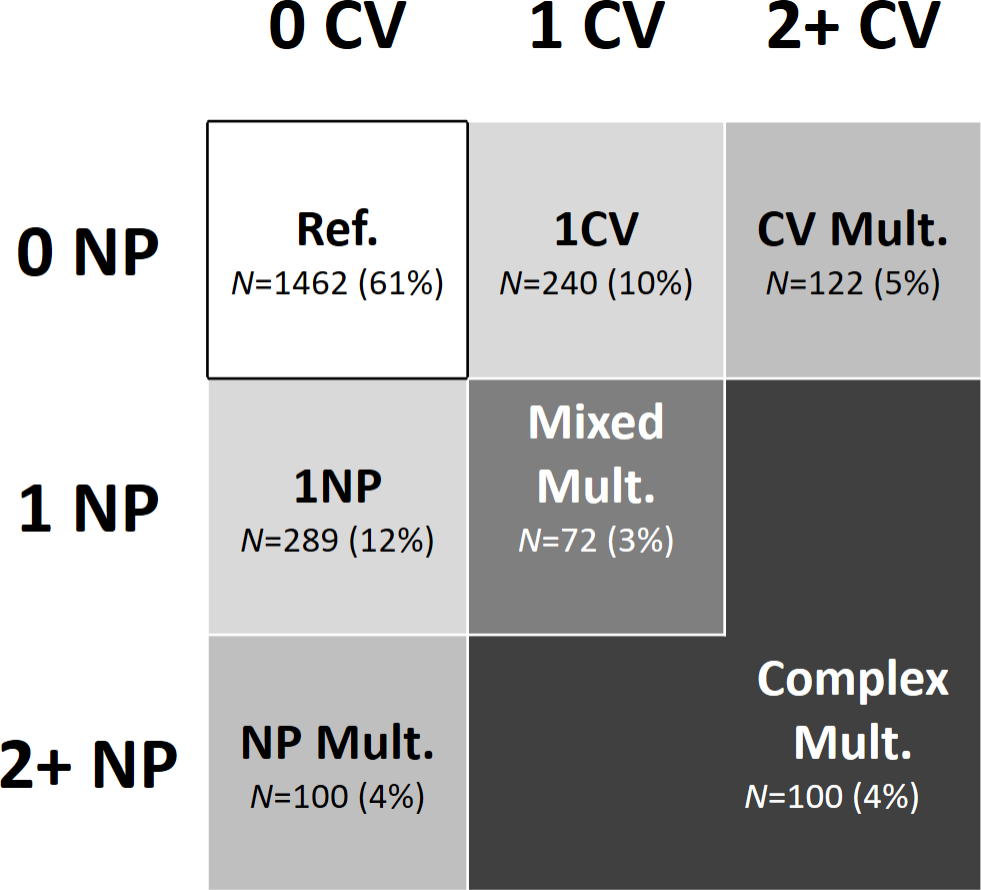
Model showing the grouping of chronic diseases. Participants were grouped by patterns of chronic diseases; that is, by number of CV and NP diseases. CV, cardiovascular; Mult., multimorbidity; NP, neuropsychiatric; Ref., reference group.

We grouped participants into 7 mutually exclusive clinical patterns based on the presence of 0, 1, or 2 or more CV or NP diseases (**Fig 2**): (1) no CV/NP diseases (reference pattern), (2) 1 CV disease, (3) 2 or more CV diseases (CV multimorbidity), (4) 1 NP disease, (5) 2 or more NP diseases (NP multimorbidity), (6) 1 CV and one NP disease (mixed multimorbidity), (7) 2 or more CV or NP diseases, accompanied by at least 1 other NP or CV disease (complex multimorbidity). The reference group may also include participants with 1 or more chronic disorders other than CV and NP diseases (see **S1 Table** for more details). Our hypothesis is that CV and NP multimorbidity has a group effect on the outcomes that goes beyond the simple count of diseases and that exceeds the effect of generic multimorbidity (2 or more diseases, not including CV and NP diseases). This is the rationale for including participants with multimorbidity in the control group. Moreover, studying the impact of mixed and complex multimorbidity may also provide information on the effect of the number and type of diseases in participants who have ≥2 CV and/or NP diseases. For the present study, information on chronic diseases was available at baseline and at the 3- and 6-year follow-ups.

### Functional decline

Mobility was assessed with walking speed and independence as the ability to carry out activities of daily living (ADL). Changes in mobility and dependence were the outcomes of the present study. To assess walking speed, participants were asked to walk 6 meters at their usual speed, or alternatively, 2.4 meters. The shorter distance was used when participants reported that they walked slowly or when the assessment was carried out in restricted spaces, such as in homes or institutions. Walking speed was reported in meters/second. The ADL score was obtained by summing the number of intact domains from the following list: bathing, dressing, toileting, continence, transferring from bed, and eating. Both walking speed and ADL were assessed at baseline and at each follow-up for up to 9 years.

### Covariates

Participants’ demographics (i.e., age, sex, and education) were collected through a questionnaire administered by the nurse [19]. Educational attainment was categorised as elementary, high school, and university or higher. Living arrangements (i.e., living in the community or in an institution) were registered. Body mass index (BMI) was obtained by dividing the participants’ weight by their squared height (kg/m^2^). A BMI <18.5 kg/m^2^ was considered indicative of malnutrition, in accordance with the recommendations of the European Society for Clinical Nutrition and Metabolism [21]. The total number of medications was obtained by counting the number of medications used at the moment of the assessment, coded in accordance with their Anatomical Therapeutic Chemical (ATC) classification. In the present study, institutionalization, malnutrition, and number of medications were used as global measures and proxies of disease severity. Data on BMI, living arrangements, and medications were available at baseline and at the 3- and 6-year follow-ups. Vital status and date of death were obtained from the Swedish National Cause of Death Register and linked to SNAC-K data.

### Statistical analyses

In a first analysis, trajectories of walking speed and ADL over 9 years were obtained with multilevel mixed-effect linear regression models. The clinical patterns at baseline were the exposures, and the analysis was adjusted for baseline age, sex, education, malnutrition, institutionalization, and number of medications. The reference group consisted of participants free from CV and NP diseases. Follow-up time was modelled through unrestricted cubic splines with 3 knots (at 0, 3, and 6 years). This analysis assessed how well the clinical patterns predicted functional decline at the individual level. In a second analysis, the association between clinical patterns and functional decline at the group level was estimated through mixed-effect regression models, in which clinical patterns and covariates that might change with time (i.e., number of medications, malnutrition, and institutionalization) were all included as time varying. This analysis assessed the cross-sectional association between clinical patterns and outcomes, considering the evolution of the exposure over time and taking intra- and interindividual variability into account. Interactions between clinical patterns and sex and age were tested, and stratified analyses were carried out. A *p*-value of <0.05 was considered statistically significant in all analyses. All analyses were carried out with Stata 14.0 (StataCorp LP) for Microsoft Windows. Compared with the original protocol (**S1 Study Protocol**), which included functional decline and mortality as outcomes, we later decided to focus our analyses on functional decline only, including walking speed (and not only disability) as outcome. Compared with the original plan, we also decided to model our outcomes through linear-mixed models instead of Cox regressions, in order to get the most from the repeated measures of both walking speed and ADL.

### Sensitivity analyses

First, to investigate the role played by individual diseases in the robustness of our results, we tested the association between the number of CV and NP diseases and functional decline, excluding participants affected by the most common diseases, one disease at a time. Second, as death and dropout during follow-up may not have been at random, we repeated the analyses, excluding those who died or dropped out during follow-up. Third, incontinence occurs more frequently in females than in males. To check whether this difference had biased any estimation, we repeated the analyses, excluding incontinence from the ADL count.

## Results

The mean age of the 2,385 participants was 73 years, and 65% were females (**Table 1**). As shown in **Table 2**, ischemic heart disease (13%), heart failure (7%), and atrial fibrillation (7%) were the 3 most frequent CV diseases at baseline. Depression and mood disorders (8%), dementia (6%), and cerebrovascular diseases (5%) were the 3 most frequent NP diseases. Chronic disease was more prevalent in participants ≥75 years than in younger participants. In the reference group (participants without CV and/or NP disease), 6% of participants were not affected by any disease, 17% had 1 disease, and 77% had 2 or more diseases. Chronic kidney disease (25%), osteoarthritis (13%), and thyroid diseases (9%) were the most common chronic diseases (**S1 Table**).

**Table 1:** Sample characteristics at baseline by patterns of CV and NP chronic diseases.

	No CV/NP diseases	1 CV disease	CV multim.	1 NP disease	NP multim.	Mixed multim.	Complex multim.	Total *N* = 2,385
***DEMOGRAPHICS***								
Sex (female; *N and %*)	944 (64.6)	129 (53.8)	70 (57.4)	206 (71.3)	83 (83.0)	47 (66.2)	65 (65.0)	**1,544 (64.8)**
Age (years; *mean ± SD*)	70.4 ± 9.5	76.7 ± 9.5	80.3 ± 8.4	73.7 ± 11.1	76.7 ± 10.3	79.2 ± 8.9	81.4 ± 9.3	**72.9 ± 10.3**
Living in institution (*N and %*)	2 (0.1)	2 (0.8)	2 (1.6)	14 (4.8)	17 (17.0)	5 (7.0)	14 (14.0)	**56 (2.4)**
EDUCATION*								
Elementary (*N and %*)	171 (11.7)	43 (17.9)	27 (22.0)	52 (18.0)	14 (14.0)	17 (23.9)	20 (20.0)	**344 (14.4)**
High school (*N and %*)	700 (47.9)	119 (49.6)	65 (53.3)	135 (49.7)	50 (50.0)	38 (53.5)	57 (57.0)	**1,164 (48.8)**
University or more (*N and %*)	591 (40.4)	77 (32.1)	30 (24.6)	98 (33.9)	35 (35.0)	15 (21.0)	21 (21.0)	**867 (36.4)**
***CLINICAL AND FUNCTIONAL ASSESSMENT***								
Malnutrition** (*N and %*)	22 (1.5)	4 (1.7)	1 (0.8)	5 (1.7)	1 (1.0)	2 (2.8)	4 (4.0)	**39 (1.6)**
*N* diseases ^¶^ (*mean ± SD*)	2.8 ± 1.7	4.6 ± 1.8	6.8 ± 2.2	4.1 ± 1.8	5.7 ± 1.9	5.7 ± 2.0	7.9 ± 2.0	**3.8 ± 2.3**
*N* medications (*mean ± SD*)	2.5 ± 2.5	5.2 ± 3.1	6.4 ± 3.7	4.1 ± 2.8	6.4 ± 3.7	5.5 ± 2.7	7.7 ± 3.8	**3.7 ± 3.2**

*9 missing values for education.

**Malnutrition defined as BMI < 18.5 kg/m^2^.

^*¶*^Number of diseases out of 60 different conditions, including CV and NP diseases, as grouped by Calderón-Larrañaga et al. [20].

Abbreviations: BMI, body mass index; CV, cardiovascular; multim., multimorbidity; *N*, number; NP, neuropsychiatric; SD, standard deviation.

**Table 2:** P/100 of CV and NP chronic diseases* and clinical patterns at baseline.

	*Total* *N = 2,385*		*<75 years* *N = 1,394 (58%)*		*75+ years* *N = 991 (42%)*	
	*N*	*P/100 (95% CI)*	*N*	*P/100 (95% CI)*	*N*	*P/100 (95% CI)*
***CV Diseases***						
*Ischemic heart disease*	312	13.1 (11.8–14.5)	102	7.3 (6.0–8.8)	211	21.2 (18.8–24.0)
*Heart failure*	176	7.4 (6.4–8.5)	27	1.9 (1.3–2.8)	149	15.0 (12.9–17.4)
*Atrial fibrillation*	168	7.1 (6.0–8.1)	44	3.1 (2.3–4.2)	124	12.5 (10.6–14.7)
*Other CV diseases*	55	2.3 (1.8–3.0)	16	1.1 (0.7–1.8)	39	3.9 (2.9–5.3)
*Cardiac valve diseases*	50	2.1 (1.6–2.8)	16	1.1 (0.7–1.9)	34	3.4 (2.5–4.8)
*Bradycardias and conduction disorders*	30	1.3 (1.0–1.9)	9	0.6 (0.3–1.2)	21	2.1 (1.4–3.2)
*Peripheral artery disease*	26	1.1 (1.0–1.6)	11	0.8 (0.4–1.4)	15	1.5 (0.9–2.5)
***NP DISEASES***						
*Depression and mood disorders*	200	8.4 (7.3–9.6)	109	7.8 (6.5–9.3)	91	9.2 (7.6–11.1)
*Cerebrovascular disease*	140	5.9 (5.0–6.9)	39	2.8 (2.0–3.8)	101	10.2 (8.5–12.2)
*Dementia*	108	4.5 (3.8–5.5)	10	0.7 (0.4–1.3)	99	10.1 (2.3–12.0)
*Neurotic, stress-related, and somatoform disorders*	73	3.1 (2.4–3.8)	37	2.7 (1.9–3.6)	36	3.6 (2.6–5.0)
*Migraine and facial pain syndromes*	50	2.1 (1.6–2.8)	38	2.7 (2.0–3.7)	12	1.2 (0.7–2.1)
*Other neurological disorders*	42	1.8 (1.3–2.4)	18	1.3 (0.8–2.0)	24	2.4 (1.6–3.6)
*Peripheral neuropathy*	36	1.5 (1.1–2.1)	14	1.0 (0.6–1.7)	22	2.2 (1.5–3.4)
*Other psychiatric and behavioral disorders*	35	1.5 (1.1–2.0)	18	1.3 (0.8–2.0)	17	1.7 (1.1–2.7)
*Parkinson and parkinsonism*	29	1.2 (1.0–1.7)	9	0.6 (0.3–1.2)	20	2.0 (1.3–3.1)
*Epilepsy*	14	0.6 (0.0–1.0)	7	0.5 (0.2–1.0)	7	0.7 (0.3–1.5)
*Schizophrenia and delusional disorders*	10	0.4 (0.0–0.8)	4	0.2 (0.1–0.8)	6	0.6 (0.3–1.3)
*Multiple sclerosis*	2	0.1 (0.0–0.2)	2	0.1 (0.0–0.6)	0	0 (-)
***CLINICAL PATTERNS***						
*No CV/NP diseases*	1,462	61.3 (60.3–62.3)	1,023	73.3 (71.0–75.6)	439	44.2 (41.2–47.4)
*1 CV disease*	240	10.0 (8.9–11.3)	101	7.2 (5.9–8.7)	139	14.0 (12.0–16.3)
*CV multimorbidity*	122	5.1 (4.3–6.1)	31	2.2 (1.6–3.0)	91	9.2 (7.5–11.1)
*1 NP disease*	289	12.1 (10.9–13.5)	156	11.1 (9.6–13.0)	133	13.4 (11.4–15.6)
*NP multimorbidity*	100	4.2 (3.5–5.1)	43	3.1 (2.3–4.1)	57	5.8 (4.5–7.4)
*Mixed multimorbidity*	72	3.0 (2.4–3.8)	20	1.4 (0.9–0.2)	52	5.2 (4.0–6.8)
*Complex multimorbidity*	100	4.2 (3.5–5.1)	20	1.4 (0.9–2.2)	80	8.1 (6.5–9.9)

* Details on the definition of listed chronic diseases are reported in S2 Text.

Abbreviations: CV, cardiovascular; NP, neuropsychiatric; P/100, proportion per 100.

**Fig 3:**
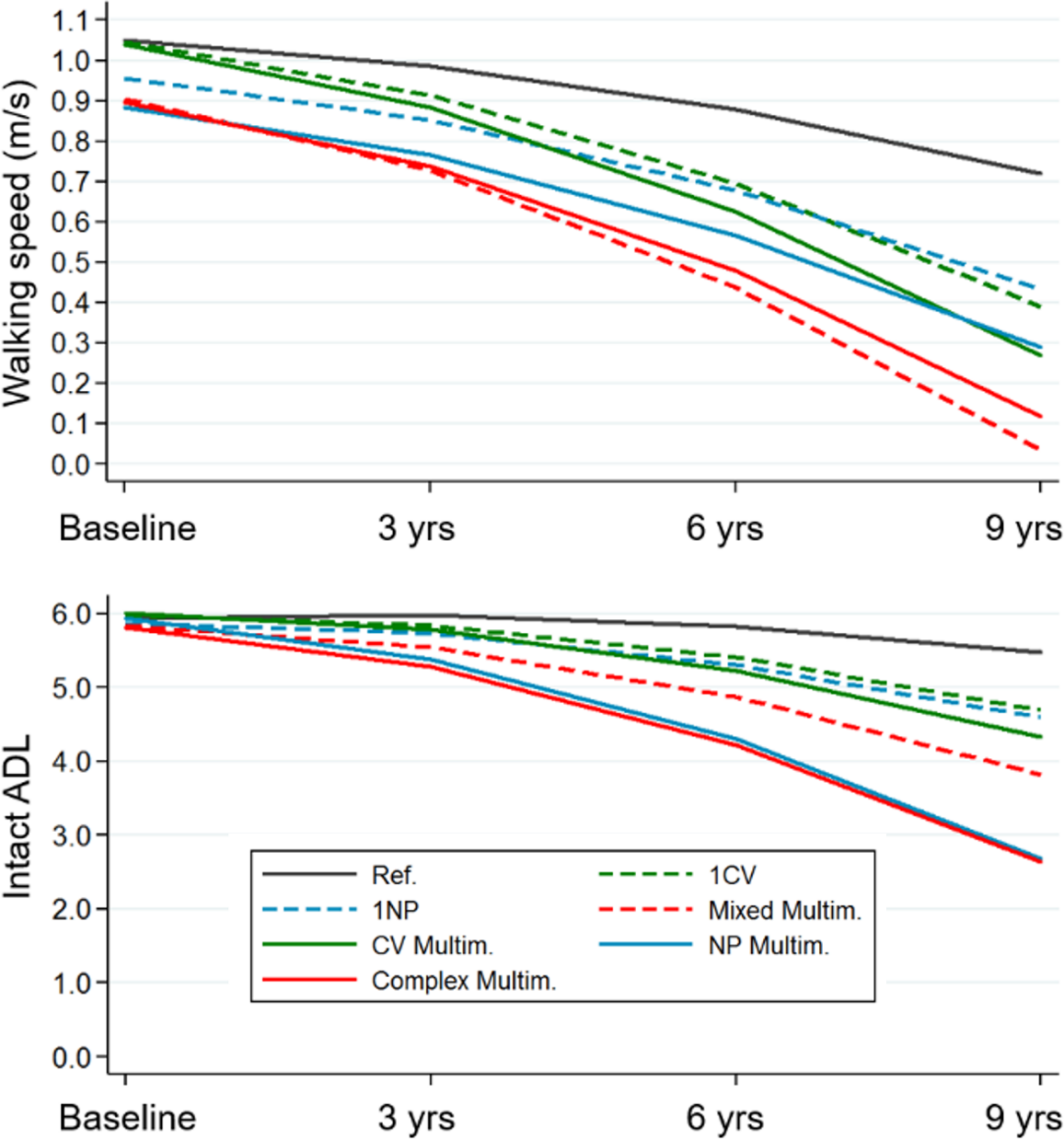
Trajectories of walking speed and ADL impairment over 9 years by clinical patterns. Trajectories derived from multilevel mixed-effect linear regression models adjusted for baseline age, sex, education, malnutrition, institutionalization, and number of medications. Reference group: participants free from cardiovascular and/or neuropsychiatric diseases. ADL, activities of daily living; CV, cardiovascular; Multim., multimorbidity; NP, neuropsychiatric; Ref., reference group.

**Fig 3** depicts the trajectories of walking speed and ADL over time. Participants with multiple NP diseases, whether or not they combined with CV diseases, had the steepest decline in both walking speed and ADL. The rate of decline of participants with CV multimorbidity was significantly higher than that of the control group (participants free from CV and NP diseases), but only in walking speed. After 9 years, participants with multiple CV and NP diseases had the most meaningful deterioration in walking speed (up to 0.7 m/s, *p* < 0.001) and ADL (up to 3 ADL, *p* < 0.001), as compared with the reference group.

**Fig 4:**
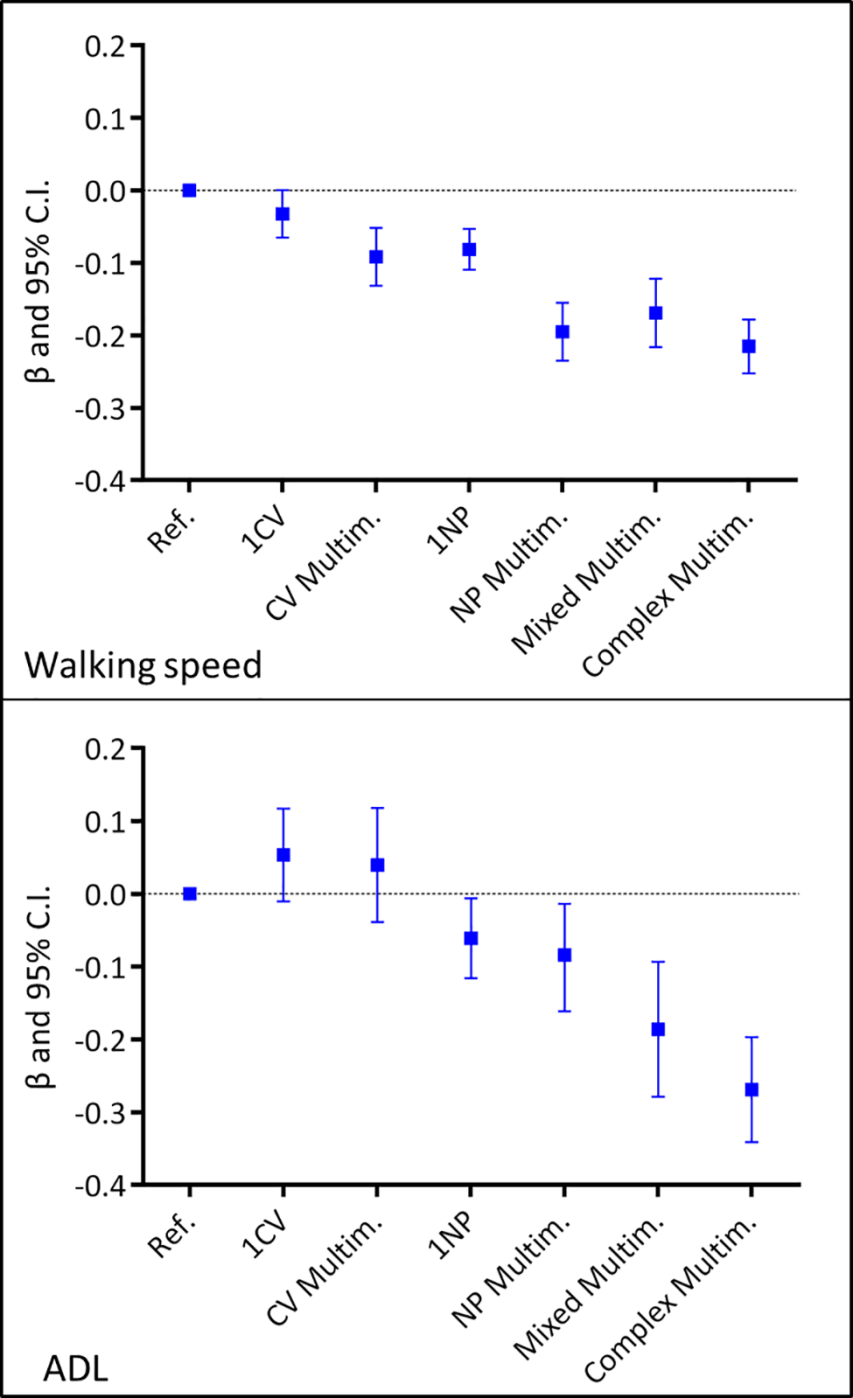
Association of chronic disease patterns with walking speed and number of intact ADL based on 6-year repeated measures of disease patterns, covariates, and outcomes. Multilevel mixed-effect linear regression models adjusted for age, sex, education, malnutrition, institutionalization, and number of medications. The exposure (the patterns of chronic diseases), the time-varying covariates (institutionalization, malnutrition, and number of medications), and the outcomes were introduced in the models as repeated measures. Reference group: participants free from CV/NP diseases. ADL, activities of daily living; CI, confidence interval; CV, cardiovascular; Multim., multimorbidity; NP, neuropsychiatric; Ref., reference group.

**Fig 4** shows the association between the clinical patterns and functional decline at the group level, considering both the clinical patterns and the covariates as time varying. Having 1 CV disease was negatively associated with walking speed, but the association was of marginal significance (β = −0.03; 95% confidence interval [CI] −0.07–0.00). All other clinical patterns were significantly negatively associated with walking speed. The strongest associations were with NP multimorbidity (β = −0.20; 95% CI −0.24–−0.16) and complex multimorbidity (β = −0.21; 95% CI −0.25–−0.18). All clinical patterns that included at least 1 NP disease were negatively associated with ADL. Mixed multimorbidity (β = −0.20; 95% CI −0.30–−0.11) and complex multimorbidity (β = −0.27; 95% CI −0.34–−0.19) had the strongest associations. Notably, 1 of 4 participants had at least 1 NP disease. In general, participants with the most complex clinical patterns had 5%–20% lower functioning than those in the reference group.

The interaction and stratified analyses showed that older age and female sex generally tended to enhance the negative association between the clinical patterns and the outcomes (**S2 and S3 Tables**). In a sensitivity analysis, the exclusion of participants with the most prevalent CV and NP diseases (**S1 Fig**) did not change the strength of the association between the number of CV and NP diseases and functional decline. The results of further sensitivity analyses were consistent with the results of our main analyses.

## Discussion

This study showed that patterns of CV and NP morbidity have different associations with functional decline in older adults over time. The pattern that included multiple CV and NP diseases was associated with the worst trajectory. NP diseases, alone or in association, are prevalent and major correlates of functional decline. Isolated CV multimorbidity was only associated with changes in mobility. In general, the combination of multiple CV and NP diseases (mixed and complex multimorbidity) was associated with 5%–20% reduced functioning than the reference group (people free from CV and NP diseases but with a high prevalence of generic multimorbidity).

A number of our results were consistent with those of previous studies. First, both CV and NP diseases were the most prevalent diseases. They also emerged as independent predictors of poor function in older adults, and NP diseases were stronger predictors than CV diseases [9]. Second, the coexistence of NP diseases boosted the association of each individual condition with functional decline, which suggests the existence of a biological interaction among NP diseases [22,23]. Third, when impairments in different systems coexist (as in mixed and complex multimorbidity), the association between the clinical patterns and the outcomes surpasses the sum of the individual associations with the outcomes and also exceeds the effect of generic multimorbidity [24,25].

Three studies have investigated the impact of multimorbidity patterns on disability, but none has looked at the impact on physical performance. Jackson et al. reported that CV and NP patterns—but not musculoskeletal pattern—were the main determinants of dependence in personal and instrumental ADL in a sample of 7,270 older women from the Australian Longitudinal Study on Women’s Health [22]. Quiñones et al. showed that a pattern of multimorbidity that included depressive symptoms, arthritis, and hypertension was associated with greater disability than other patterns in a sample of 8,782 older adults participating in the Health and Retirement Study [2]. The same group showed that in a sample of 4,017 older Medicare beneficiaries, combinations of somatic and mental diseases, in particular cognitive impairment and dementia, were associated with greater incident disability than combinations of somatic conditions only [25].

Only indirect comparisons can be made between our study and previous studies. In addition to differences in the sample selection, follow-up duration, and data collection, a number of aspects of our study require special attention: (1) unlike in previous studies, in which ad hoc lists of diseases were used, we relied on a comprehensive, clinically driven list of chronic diseases, for which the proof of concept was recently shown in the SNAC-K population [20]. (2) Our patterns of CV and NP diseases were not built with a data-driven procedure (e.g., factor analysis or prevalence), and we included all CV and NP diseases deemed chronic in the older population by Calderón-Larrañaga et al. [20]. (3) As opposed to previous studies, to test the association between disease patterns and functional status at the group level, we also took the within-individual, time-varying nature of multimorbidity into consideration and accounted for time variation in covariates (e.g., medication use and institutionalization). People age at different rates, and as a consequence, the development of multimorbidity follows different trajectories. Capturing the dynamic nature of multimorbidity in clinical and epidemiological studies is a scientific priority [6, 20].

Aging is a complex and multifaceted process, and underlying it are a large number of biological deficits that ultimately affect people’s resilience to external stressors [1,2]. The World Health Organization has defined healthy aging as the “process of developing and maintaining the functional ability that enables well-being in older age” [26]. Given the wide heterogeneity of clinical phenotypes in older people, it is preferable to use global measures of functioning to capture and assess their health status [27,28]. The National Institute for Health and Care Excellence (NICE) issued the first guidelines for the clinical assessment and management of multimorbidity in 2016. According to these guidelines, frailty and functional decline are both a component of multimorbidity and a condition that should be taken into account and assessed when managing the health and care of older adults with multimorbidity. [29]. In the present study, we chose walking speed and ADL as indicators of function in older adults. Slow walking speed is a strong and independent predictor of disability and a measure of physical frailty [28]. As shown by Santoni et al., slow walking speed and dependency in ADL optimally discriminate older adults’ health across different ages. Walking speed begins to decrease during the sixth decade of life, whereas ADL impairment tends to become evident only during the eighth decade [30]. In the present study, the effect of CV diseases and CV multimorbidity was more evident on mobility than on independence. Adequate walking speed and ADL both involve several systems and organs, and the integrity of mental, musculoskeletal, and CV functions are equally required [28,31]. However, unlike for ADL, walking—especially walking at an adequate speed—requires a certain level of CV fitness. The lack of such fitness due to the presence of 1 or more CV diseases might explain the selective association between CV diseases and walking speed [32]. NP conditions, on the other hand, were associated with both measures of functional decline. In particular, there was a clear dose-response association between walking speed and the higher number of coexisting NP conditions. Severe NP diseases, such as dementia and cerebrovascular disease (e.g., stroke), have a direct impact on quality of life and independence and are extremely burdensome, even in the presence of an intact CV system [9]. This finding has direct clinical implications. First, general practitioners and specialists should assess mobility in patients with multiple NP diseases more often than in others because of the steeper functional decline they are expected to experience. Similarly, physicians should consider the potential negative effect of medications for NP diseases on functional decline. Second, patients with multiple NP diseases and their caregivers are often told about the memory decline or NP symptoms they could develop but not about potential functional decline. Enhancing their awareness of this eventuality may help empower them to postpone or minimise the detrimental effects of diseases. For example, patients could ask for interventions to slow functional decline in the early stages. All these issues have clear public health implications. Finally, consistent with previous findings, the co-occurrence of diseases involving different systems (i.e., CV and NP) was associated with slower walking speed and greater ADL dependency. However, the strength of this association largely overlaps with that of NP multimorbidity.

Some limitations of this study need to be mentioned. First, studying the combined effect of different diseases belonging to the same body system (i.e., CV and NP) prevented us from looking at the differential impact of individual conditions. For example, we might expect a different prognosis for dementia and depression. However, in sensitivity analyses, excluding those with the most prevalent diseases, one disease at a time, did not change the association between the number of CV and NP diseases and the outcomes. This suggests that our hypothesis that homogenous patterns of multimorbidity convey an overall group-specific effect is plausible. Similar findings have been reported previously [7]. Second, we did not take the severity of diseases into account. However, we adjusted our analyses for a number of factors (i.e., malnutrition, institutionalization, and number of medications) that can be considered indirect proxies of disease severity and, at the same time, indicators of other comorbid conditions not explicitly accounted for. The same approach used to estimate the severity of single diseases might not work in the presence of multimorbidity. For this reason, using global and multiple proxies of complexity is warranted [33]. In addition, when we tested the overall association between disease patterns and functional status, these covariates were analyzed as time varying. Third, the study of the prognosis of heterogeneous and less prevalent disease patterns (i.e., mixed and complex multimorbidity) may have introduced some noise into our analyses. However, the strength and precision of the associations suggests that our hypothesis regarding the dose-response effect and interaction between multiple CV and NP diseases is plausible. Fourth, the SNAC-K sample selection (73% participation rate) might have affected the generalizability of the present study. In particular, nonparticipants had shorter survival (see S1 Text), which may mean that both multimorbidity and poor physical function were underestimated in the analyses, partially blunting the associations we observed between the clinical patterns and functional decline. Fifth, the exclusion of participants with less than 2 assessments may have led to an underestimation of the association between clinical patterns and walking speed and ADL, but only at baseline. However, as we were interested in long-term trajectories of functional decline, we believe that this exclusion criterion was reasonable. Notably, in the analyses that included all SNAC-K participants, the baseline association between clinical patterns and the outcomes was stronger than the baseline association in the main analysis, but rates of decline over the follow-up period were similar. Finally, as shown in previous studies, SNAC-K participants are relatively fit and wealthy. This might further limit the generalizability of our results to other groups of older adults.

### Conclusion

In conclusion, our findings indicate that CV and NP multimorbidity had different associations with older adults’ health. NP, mixed, and complex multimorbidity were associated with the highest burden of functional decline, even with respect to participants with high prevalence of generic multimorbidity. CV diseases were less strongly associated with functional decline than were NP diseases; CV diseases were only associated with declines in mobility. Moreover, none of the diseases drove the association between the group to which it belonged and functional decline. This finding corroborates the idea that identifying patterns of chronic diseases can assist in managing the health and care of people with multimorbidity by providing useful prognostic information, highlighting clinical priorities, steering therapeutic interventions, and helping physicians counsel patients and their families.

## Supporting information

S1 Checklist STROBE checklist. (DOCX)

S1 Table Distribution of non-CV and non-NP chronic conditions at baseline in the reference group. CV, cardiovascular; NP, neuropsychiatric.
(DOCX)

S2 Table Main analysis stratified by sex and age. Outcome: walking speed.
(DOCX)

S3 Table Main analysis stratified by sex and age. Outcome: ADL. ADL, activities of daily living.
(DOCX)

S1 Fig Association of the row number of CV and NP diseases with (a) walking speed and (b) ADL impairment, after excluding from the sample, one at a time, groups of participants suffering from the most frequent CV and NP diseases. ADL, activities of daily living; CV, cardiovascular; NP, neuropsychiatric.
(DOCX)

S1 Text Focus on the participation rate in SNAC-K. SNAC-K, Swedish National study of Aging and Care in Kungsholmen.
(DOCX)

S2 Text Descriptors of ICD-10 codes included and excluded in each CV and NP chronic disease category. CV, cardiovascular; ICD-10, International Classification of Diseases, 10th revision; NP, neuropsychiatric.
(DOCX)

S1 Study Protocol Study Protocol. (DOCX)

## Abbreviations

ADL: activities of daily living
ATC: Anatomical Therapeutic Chemical
BMI: body mass index
CI: confidence interval
CV: cardiovascular
DALY: disability-adjusted life year
ICD-10: International Classification of Diseases, 10th revision
N: number
NICE: National Institute for Health and Care Excellence
NP: neuropsychiatric
P/100: proportion per 100
SD: standard deviation
SNAC-K: Swedish National study of Aging and Care in Kungsholmen
