# Supplementary material for: Trajectories of functional decline in older adults with neuropsychiatric and cardiovascular multimorbidity: A Swedish cohort study
Source: PLoS Med. 2018 Mar 6;15(3):e1002503. doi: 10.1371/journal.pmed.1002503 (PMC5839531; doi:10.1371/journal.pmed.1002503)
Supplement: S1 Table — CV, cardiovascular; NP, neuropsychiatric. (DOCX) [file pmed.1002503.s002.docx]

**Table S1. Distribution of non-CV and non-NP chronic conditions, at baseline, in the reference group (N=1462; no CV and/or no NP diseases).**

| **Condition** | **Number and prevalence (%)** |
| --- | --- |
| 0 diseases  1 disease  2+ diseases | 89 (6)  247(17)  1126 (77) |
| Chronic kidney disease | 360 (25) |
| Osteoarthritis and deg. joint diseases | 183 (13) |
| Thyroid diseases | 129 (9) |
| Colitis and related diseases | 111 (8) |
| Solid neoplasm | 102 (7) |
| Anaemia | 85 (6) |
| Diabetes | 73 (5) |
| Glaucoma | 65 (5) |
| Cataract | 69 (5) |
| COPD | 45 (3) |
