# Supplementary material for: Trajectories of functional decline in older adults with neuropsychiatric and cardiovascular multimorbidity: A Swedish cohort study
Source: PLoS Med. 2018 Mar 6;15(3):e1002503. doi: 10.1371/journal.pmed.1002503 (PMC5839531; doi:10.1371/journal.pmed.1002503)
Supplement: S2 Table — Outcome: walking speed. (DOCX) [file pmed.1002503.s003.docx]

**Table S2**. Main analysis stratified by sex and age. Outcome: **walking speed**

| **Disease Pattern** | **<75 years old** | | | **75+ years old** | | |  |
| --- | --- | --- | --- | --- | --- | --- | --- |
|  | **Beta** | **95% C.I.** | | **Beta** | **95% C.I.** | | **P for Interaction** |
| Ref. | 0 | - | - | 0 | - | - | - |
| 1 CV dis. | -0.01 | -0.05 | 0.03 | **-0.06** | **-0.10** | **-0.01** | 0.499 |
| CV Multim. | **-0.08** | **-0.15** | **-0.01** | **-0.10** | **-0.15** | **-0.05** | 0.962 |
| 1 NP Dis. | **-0.03** | **-0.07** | **-0.01** | **-0.12** | **-0.16** | **-0.08** | <0.001 |
| NP Multim. | **-0.13** | **-0.18** | **-0.07** | **-0.24** | **-0.30** | **-0.18** | 0.005 |
| Mixed Multim. | -0.05 | -0.13 | 0.02 | **-0.23** | **-0.29** | **-0.17** | <0.001 |
| Complex Multim. | **-0.15** | **-0.22** | **-0.08** | **-0.23** | **-0.28** | **-0.18** | 0.207 |

| **Disease Pattern** | **Males** | | | **Females** | | |  |
| --- | --- | --- | --- | --- | --- | --- | --- |
|  | **Beta** | **95% C.I.** | | **Beta** | **95% C.I.** | | **P for Interaction** |
| Ref. | 0 | - | - | 0 | - | - | - |
| 1 CV dis. | -0.01 | -0.05 | 0.04 | **-0.05** | **-0.10** | **-0.02** | 0.027 |
| CV Multim. | **-0.06** | **-0.13** | **-0.01** | **-0.11** | **-0.16** | **-0.06** | 0.044 |
| 1 NP Dis. | **-0.08** | **-0.13** | **-0.03** | **-0.08** | **-0.11** | **-0.04** | 0.928 |
| NP Multim. | **-0.21** | **-0.29** | **-0.13** | **-0.19** | **-0.24** | **-0.15** | 0.620 |
| Mixed Multim. | **-0.12** | **-0.20** | **-0.04** | **-0.19** | **-0.25** | **-0.14** | 0.082 |
| Complex Multim. | **-0.20** | **-0.27** | **-0.13** | **-0.22** | **-0.27** | **-0.18** | 0.636 |

Models adjusted for: age (if necessary), sex (if necessary), education, malnutrition, institutionalization, number of medications.
