# Supplementary material for: Trajectories of functional decline in older adults with neuropsychiatric and cardiovascular multimorbidity: A Swedish cohort study
Source: PLoS Med. 2018 Mar 6;15(3):e1002503. doi: 10.1371/journal.pmed.1002503 (PMC5839531; doi:10.1371/journal.pmed.1002503)
Supplement: S3 Table — Outcome: ADL. ADL, activities of daily living. (DOCX) [file pmed.1002503.s004.docx]

**Table S3**. Analysis stratified by sex and age. Outcome: **ADL**.

| **Disease Pattern** | **<75 years old** | | | **75+ years old** | | |  |
| --- | --- | --- | --- | --- | --- | --- | --- |
|  | **Beta** | **95% C.I.** | | **Beta** | **95% C.I.** | | **P for Interaction** |
| Ref. | 0 | - | - | 0 | - | - | - |
| 1 CV dis. | -0.02 | -0.05 | 0.03 | 0.03 | -0.08 | 0.15 | 0.892 |
| CV Multim. | 0.02 | -0.03 | 0.08 | 0.01 | -0.12 | 0.14 | 0.254 |
| 1 NP Dis. | -0.03 | -0.06 | 0.01 | **-0.11** | **-0.21** | **-0.01** | 0.005 |
| NP Multim. | -0.01 | -0.07 | 0.03 | -0.13 | -0.28 | 0.01 | 0.001 |
| Mixed Multim. | 0.01 | -0.05 | 0.08 | **-0.31** | **-0.46** | **-0.15** | 0.001 |
| Complex Multim. | **-0.16** | **-0.23** | **-0.10** | **-0.32** | **-0.45** | **-0.20** | 0.001 |

| **Disease Pattern** | **Males** | | | **Females** | | |  |
| --- | --- | --- | --- | --- | --- | --- | --- |
|  | **Beta** | **95% C.I.** | | **Beta** | **95% C.I.** | | **P for Interaction** |
| Ref. | 0 | - | - | 0 | - | - | - |
| 1 CV dis. | 0.01 | -0.06 | 0.03 | 0.05 | -0.07 | 0.16 | 0.846 |
| CV Multim. | 0.02 | -0.04 | 0.09 | 0.05 | -0.08 | 0.17 | 0.318 |
| 1 NP Dis. | 0.03 | -0.06 | 0.01 | -0.09 | -0.20 | 0.02 | 0.054 |
| NP Multim. | -0.10 | -0.21 | 0.01 | -0.09 | -0.19 | 0.01 | 0.676 |
| Mixed Multim. | **-0.11** | **-0.06** | **-0.08** | **-0.18** | **-0.31** | **-0.05** | 0.425 |
| Complex Multim. | **-0.17** | **-0.23** | **-0.11** | **-0.31** | **-0.41** | **-0.21** | 0.005 |

Models adjusted for: age (if necessary), sex (if necessary), education, malnutrition, institutionalization, number of medications.
