# Supplementary material for: Trajectories of functional decline in older adults with neuropsychiatric and cardiovascular multimorbidity: A Swedish cohort study
Source: PLoS Med. 2018 Mar 6;15(3):e1002503. doi: 10.1371/journal.pmed.1002503 (PMC5839531; doi:10.1371/journal.pmed.1002503)
Supplement: S1 Fig — Association of the row number of CV and NP diseases with (a) walking speed and (b) ADL impairment, after excluding from the sample, one at a time, groups of participants suffering from the most frequent CV and NP diseases. ADL, activities of daily living; CV, cardiovascular; NP, neuropsychiatric. (DOCX) [file pmed.1002503.s005.docx]

**Figure S2.** Association of the row number of CV and NP diseases with (a) walking speed and (b) ADL impairment, after excluding from the sample, one at a time, groups of participants suffering from the most frequent CV and NP diseases. The “Overall” estimation represents the association between the overall number of CV or NP diseases and the outcomes.

**
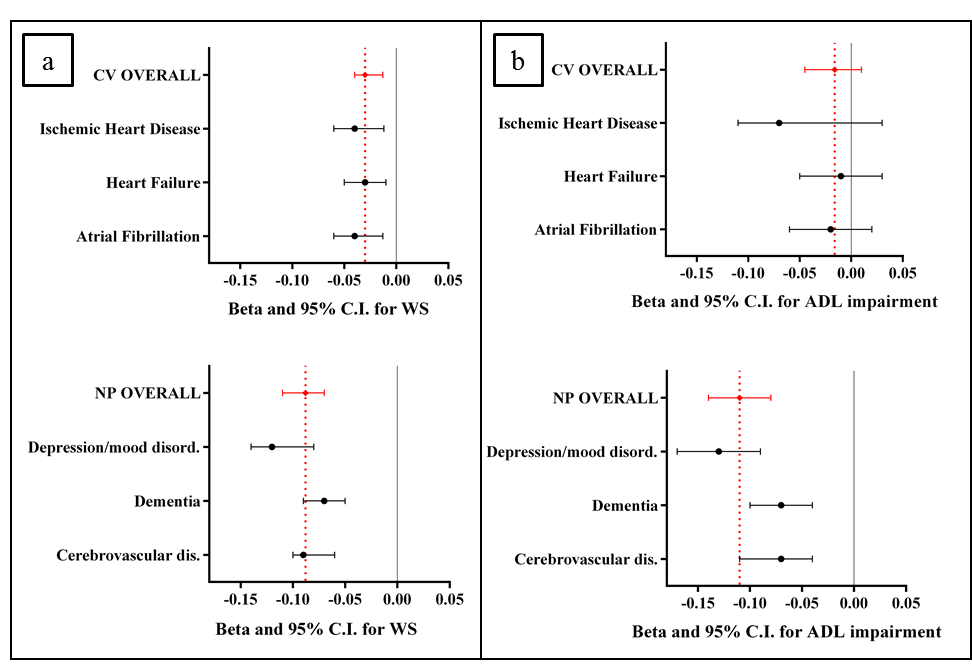
**

Models adjusted for: age, sex, education, malnutrition, institutionalization, number of medications and either number of CV or NP diseases, as appropriate.
