# Supplementary material for: Trajectories of functional decline in older adults with neuropsychiatric and cardiovascular multimorbidity: A Swedish cohort study
Source: PLoS Med. 2018 Mar 6;15(3):e1002503. doi: 10.1371/journal.pmed.1002503 (PMC5839531; doi:10.1371/journal.pmed.1002503)
Supplement: S1 Text — SNAC-K, Swedish National study of Aging and Care in Kungsholmen. (DOCX) [file pmed.1002503.s006.docx]

**S1 text**

**Focus on the participation rate in SNAC-K**

Out of 5111 people invited to participate to the Swedish National study of Aging and Care in Kungsholmen (SNAC-K), 521 were not eligible (200 died, 262 had no contact information: 59 were deaf, moved away, or were not Swedish speakers). Out of the remaining 4590, 1227 declined to participate, leaving a study population of 3363 (73% participation rate). Participation rates were above 70% in all age groups and were similar among men and women.

The proportion of people living in institutions was significantly higher among participants (6%) than among non-participants (12 people, <1%), although it is possible the latter figure is underestimated, as there were some people originally invited to participate who could not be contacted afterwards (N = 262). For the age cohorts 60 through 87, shorter time to death after the beginning of the study was associated with higher risk of non-participation.
