# Supplementary material for: Trajectories of functional decline in older adults with neuropsychiatric and cardiovascular multimorbidity: A Swedish cohort study
Source: PLoS Med. 2018 Mar 6;15(3):e1002503. doi: 10.1371/journal.pmed.1002503 (PMC5839531; doi:10.1371/journal.pmed.1002503)
Supplement: S2 Text — CV, cardiovascular; ICD-10, International Classification of Diseases, 10th revision; NP, neuropsychiatric. (DOCX) [file pmed.1002503.s007.docx]

**Descriptors of ICD-10 codes included and excluded in each CV and NP chronic disease category.**

| **ATRIAL FIBRILLATION** | |
| --- | --- |
| **Included ICD-10 codes and labels** | |
| I48 | Atrial fibrillation and flutter |
| **BRADYCARDIAS AND CONDUCTION DISEASES** | |
| **Included ICD-10 codes and labels** | |
| I441 | Atrioventricular block, second degree |
| I442 | Atrioventricular block, complete |
| I443 | Other and unspecified atrioventricular block |
| I453 | Trifascicular block |
| I455 | Other specified heart block |
| Z950 | Presence of cardiac pacemaker |
| **CARDIAC VALVE DISEASES** | |
| **Included ICD-10 codes and labels** | |
| I05 | Rheumatic mitral valve diseases |
| I06 | Rheumatic aortic valve diseases |
| I07 | Rheumatic tricuspid valve diseases |
| I08 | Multiple valve diseases |
| I091 | Rheumatic diseases of endocardium, valve unspecified |
| I098 | Other specified rheumatic heart diseases |
| I34 | Nonrheumatic mitral valve disorders |
| I35 | Nonrheumatic aortic valve disorders |
| I36 | Nonrheumatic tricuspid valve disorders |
| I37 | Pulmonary valve disorders |
| I38 | Endocarditis, valve unspecified |
| I390 | Mitral valve disorders in diseases classified elsewhere |
| I391 | Aortic valve disorders in diseases classified elsewhere |
| I392 | Tricuspid valve disorders in diseases classified elsewhere |
| I393 | Pulmonary valve disorders in diseases classified elsewhere |
| I394 | Multiple valve disorders in diseases classified elsewhere |
| Q22 | Congenital malformations of pulmonary and tricuspid valves |
| Q23 | Congenital malformations of aortic and mitral valves |
| Z952 | Presence of prosthetic heart valve |
| Z953 | Presence of xenogenic heart valve |
| Z954 | Presence of other heart-valve replacement |
| **CEREBROVASCULAR DISEASE** | |
| **Included ICD-10 codes and labels** | |
| G45 | Transient cerebral ischaemic attacks and related syndromes |
| G46 | Vascular syndromes of brain in cerebrovascular diseases |
| I60 | Subarachnoid haemorrhage |
| I61 | Intracerebral haemorrhage |
| I62 | Other nontraumatic intracranial haemorrhage |
| I63 | Cerebral infarction |
| I64 | Stroke, not specified as haemorrhage or infarction |
| I67 | Other cerebrovascular diseases |
| I69 | Sequelae of cerebrovascular disease |
| **DEMENTIA** | |
| **Included ICD-10 codes and labels** | |
| F00 | Dementia in Alzheimer disease |
| F01 | Vascular dementia |
| F02 | Dementia in other diseases classified elsewhere |
| F03 | Unspecified dementia |
| F051 | Delirium superimposed on dementia |
| G30 | Alzheimer disease |
| G31 | Other degenerative diseases of nervous system, not elsewhere classified |
| **DEPRESSION AND MOOD DISEASES** | |
| **Included ICD-10 codes and labels** | |
| F30 | Manic episode |
| F31 | Bipolar affective disorder |
| F32 | Depressive episode |
| F33 | Recurrent depressive disorder |
| F34 | Persistent mood [affective] disorders |
| F38 | Other mood [affective] disorders |
| F39 | Unspecified mood [affective] disorder |
| F412 | Mixed anxiety and depressive disorder |
| **EPILEPSY** | |
| **Included ICD-10 codes and labels** | |
| G40 | Epilepsy |
| **Excluded ICD-10 codes and labels** | |
| G405 | Special epileptic syndromes |
| **HEART FAILURE** | |
| **Included ICD-10 codes and labels** | |
| I110 | Hypertensive heart disease with (congestive) heart failure |
| I130 | Hypertensive heart and renal disease with (congestive) heart failure |
| I132 | Hypertensive heart and renal disease with both (congestive) heart failure and renal failure |
| I27 | Other pulmonary heart diseases |
| I280 | Arteriovenous fistula of pulmonary vessels |
| I42 | Cardiomyopathy |
| I43 | Cardiomyopathy in diseases classified elsewhere |
| I50 | Heart failure |
| I515 | Myocardial degeneration |
| I517 | Cardiomegaly |
| I528 | Other heart disorders in other diseases classified elsewhere |
| Z941 | Heart transplant status |
| Z943 | Heart and lungs transplant status |
| **ISCHEMIC HEART DISEASE** | |
| **Included ICD-10 codes and labels** | |
| I20 | Angina pectoris |
| I21 | Acute myocardial infarction |
| I22 | Subsequent myocardial infarction |
| I24 | Other acute ischaemic heart diseases |
| I25 | Chronic ischaemic heart disease |
| Z951 | Presence of aortocoronary bypass graft |
| Z955 | Presence of coronary angioplasty implant and graft |
| **MIGRAINE AND FACIAL PAIN SYNDROMES** | |
| **Included ICD-10 codes and labels** | |
| G43 | Migraine |
| G440 | Cluster headache syndrome |
| G441 | Vascular headache, not elsewhere classified |
| G442 | Tension-type headache |
| G443 | Chronic post-traumatic headache |
| G448 | Other specified headache syndromes |
| G50 | Disorders of trigeminal nerve |
| **MULTIPLE SCLEROSIS** | |
| **Included ICD-10 codes and labels** | |
| G35 | Multiple sclerosis |
| **NEUROTIC, STRESS-RELATED AND SOMATOFORM DISEASES** | |
| **Included ICD-10 codes and labels** | |
| F40 | Phobic anxiety disorders |
| F41 | Other anxiety disorders |
| F42 | Obsessive-compulsive disorder |
| F43 | Reaction to severe stress, and adjustment disorders |
| F44 | Dissociative [conversion] disorders |
| F45 | Somatoform disorders |
| F48 | Other neurotic disorders |
| **Excluded ICD-10 codes and labels** | |
| F430 | Acute stress reaction |
| F432 | Adjustment disorders |
| **OTHER CARDIOVASCULAR DISEASES** | |
| **Included ICD-10 codes and labels** | |
| I09 | Other rheumatic heart diseases |
| I281 | Aneurysm of pulmonary artery |
| I310 | Chronic adhesive pericarditis |
| I311 | Chronic constrictive pericarditis |
| I456 | Pre-excitation syndrome |
| I495 | Sick sinus syndrome |
| I498 | Other specified cardiac arrhythmias |
| I70 | Atherosclerosis |
| I71 | Aortic aneurysm and dissection |
| I72 | Other aneurysm and dissection |
| I790 | Aneurysm of aorta in diseases classified elsewhere |
| I791 | Aortitis in diseases classified elsewhere |
| I950 | Idiopathic hypotension |
| I951 | Orthostatic hypotension |
| I958 | Other hypotension |
| Q20 | Congenital malformations of cardiac chambers and connections |
| Q21 | Congenital malformations of cardiac septa |
| Q24 | Other congenital malformations of heart |
| Q25 | Congenital malformations of great arteries |
| Q26 | Congenital malformations of great veins |
| Q27 | Other congenital malformations of peripheral vascular system |
| Q28 | Other congenital malformations of circulatory system |
| Z958 | Presence of other cardiac and vascular implants and grafts |
| Z959 | Presence of cardiac and vascular implant and graft, unspecified |
| **Excluded ICD-10 codes and labels** | |
| I091 | Rheumatic diseases of endocardium, valve unspecified |
| I098 | Other specified rheumatic heart diseases |
| I702 | Atherosclerosis of arteries of extremities |
| **OTHER NEUROLOGICAL DISEASES** | |
| **Included ICD-10 codes and labels** | |
| B900 | Sequelae of central nervous system tuberculosis |
| D482 | Neoplasm of uncertain or unknown behaviour: Peripheral nerves and autonomic nervous system |
| G041 | Tropical spastic paraplegia |
| G09 | Sequelae of inflammatory diseases of central nervous system |
| G10 | Huntington disease |
| G11 | Hereditary ataxia |
| G12 | Spinal muscular atrophy and related syndromes |
| G13 | Systemic atrophies primarily affecting central nervous system in diseases classified elsewhere |
| G24 | Dystonia |
| G25 | Other extrapyramidal and movement disorders |
| G26 | Extrapyramidal and movement disorders in diseases classified elsewhere |
| G32 | Other degenerative disorders of nervous system in diseases classified elsewhere |
| G37 | Other demyelinating diseases of central nervous system |
| G51 | Facial nerve disorders |
| G52 | Disorders of other cranial nerves |
| G53 | Cranial nerve disorders in diseases classified elsewhere |
| G70 | Myasthenia gravis and other myoneural disorders |
| G71 | Primary disorders of muscles |
| G723 | Periodic paralysis |
| G724 | Inflammatory myopathy, not elsewhere classified |
| G728 | Other specified myopathies |
| G729 | Myopathy, unspecified |
| G73 | Disorders of myoneural junction and muscle in diseases classified elsewhere |
| G80 | Cerebral palsy |
| G81 | Hemiplegia |
| G82 | Paraplegia and tetraplegia |
| G83 | Other paralytic syndromes |
| G90 | Disorders of autonomic nervous system |
| G91 | Hydrocephalus |
| G938 | Other specified disorders of brain |
| G939 | Disorder of brain, unspecified |
| G95 | Other diseases of spinal cord |
| G99 | Other disorders of nervous system in diseases classified elsewhere |
| M471 | Other spondylosis with myelopathy |
| Q00 | Anencephaly and similar malformations |
| Q01 | Encephalocele |
| Q02 | Microcephaly |
| Q03 | Congenital hydrocephalus |
| Q04 | Other congenital malformations of brain |
| Q05 | Spina bifida |
| Q06 | Other congenital malformations of spinal cord |
| Q07 | Other congenital malformations of nervous system |
| Q760 | Spina bifida occulta |
| **Excluded ICD-10 codes and labels** | |
| G130 | Paraneoplastic neuromyopathy and neuropathy |
| G131 | Other systemic atrophy primarily affecting central nervous system in neoplastic disease |
| G251 | Drug-induced tremor |
| G254 | Drug-induced chorea |
| G256 | Drug-induced tics and other tics of organic origin |
| G510 | Bell palsy |
| G732 | Other myasthenic syndromes in neoplastic disease |
| G733 | Myasthenic syndromes in other diseases classified elsewhere |
| G734 | Myopathy in infectious and parasitic diseases classified elsewhere |
| G838 | Other specified paralytic syndromes |
| **OTHER PSYCHIATRIC AND BEHAVIORAL DISEASES** | |
| **Included ICD-10 codes and labels** | |
| F04 | Organic amnesic syndrome, not induced by alcohol and other psychoactive substances |
| F06 | Other mental disorders due to brain damage and dysfunction and to physical disease |
| F07 | Personality and behavioural disorders due to brain disease, damage and dysfunction |
| F09 | Unspecified organic or symptomatic mental disorder |
| F102 | Mental and behavioural disorders due to use of alcohol: Dependence syndrome |
| F106 | Mental and behavioural disorders due to use of alcohol: Amnesic syndrome |
| F107 | Mental and behavioural disorders due to use of alcohol: Residual and late-onset psychotic disorder |
| F112 | Mental and behavioural disorders due to use of opioids: Dependence syndrome |
| F116 | Mental and behavioural disorders due to use of opioids: Amnesic syndrome |
| F117 | Mental and behavioural disorders due to use of opioids: Residual and late-onset psychotic disorder |
| F122 | Mental and behavioural disorders due to use of cannabinoids: Dependence syndrome |
| F126 | Mental and behavioural disorders due to use of cannabinoids: Amnesic syndrome |
| F127 | Mental and behavioural disorders due to use of cannabinoids: Residual and late-onset psychotic disorder |
| F132 | Mental and behavioural disorders due to use of sedatives or hypnotics: Dependence syndrome |
| F136 | Mental and behavioural disorders due to use of sedatives or hypnotics: Amnesic syndrome |
| F137 | Mental and behavioural disorders due to use of sedatives or hypnotics: Residual and late-onset psychotic disorder |
| F142 | Mental and behavioural disorders due to use of cocaine: Dependence syndrome |
| F146 | Mental and behavioural disorders due to use of cocaine: Amnesic syndrome |
| F147 | Mental and behavioural disorders due to use of cocaine: Residual and late-onset psychotic disorder |
| F152 | Mental and behavioural disorders due to use of other stimulants, including caffeine: Dependence syndrome |
| F156 | Mental and behavioural disorders due to use of other stimulants, including caffeine: Amnesic syndrome |
| F157 | Mental and behavioural disorders due to use of other stimulants, including caffeine: Residual and late-onset psychotic disorder |
| F162 | Mental and behavioural disorders due to use of hallucinogens: Dependence syndrome |
| F166 | Mental and behavioural disorders due to use of hallucinogens: Amnesic syndrome |
| F167 | Mental and behavioural disorders due to use of hallucinogens: Residual and late-onset psychotic disorder |
| F172 | Mental and behavioural disorders due to use of tobacco: Dependence syndrome |
| F176 | Mental and behavioural disorders due to use of tobacco: Amnesic syndrome |
| F177 | Mental and behavioural disorders due to use of tobacco: Residual and late-onset psychotic disorder |
| F182 | Mental and behavioural disorders due to use of volatile solvents: Dependence syndrome |
| F186 | Mental and behavioural disorders due to use of volatile solvents: Amnesic syndrome |
| F187 | Mental and behavioural disorders due to use of volatile solvents: Residual and late-onset psychotic disorder |
| F192 | Mental and behavioural disorders due to multiple drug use and use of other psychoactive substances: Dependence syndrome |
| F196 | Mental and behavioural disorders due to multiple drug use and use of other psychoactive substances: Amnesic syndrome |
| F197 | Mental and behavioural disorders due to multiple drug use and use of other psychoactive substances: Residual and late-onset psychotic disorder |
| F50 | Eating disorders |
| F52 | Sexual dysfunction, not caused by organic disorder or disease |
| F60 | Specific personality disorders |
| F61 | Mixed and other personality disorders |
| F62 | Enduring personality changes, not attributable to brain damage and disease |
| F63 | Habit and impulse disorders |
| F68 | Other disorders of adult personality and behaviour |
| F70 | Mild mental retardation |
| F71 | Moderate mental retardation |
| F72 | Severe mental retardation |
| F73 | Profound mental retardation |
| F78 | Other mental retardation |
| F79 | Unspecified mental retardation |
| F80 | Specific developmental disorders of speech and language |
| F81 | Specific developmental disorders of scholastic skills |
| F82 | Specific developmental disorder of motor function |
| F83 | Mixed specific developmental disorders |
| F84 | Pervasive developmental disorders |
| F88 | Other disorders of psychological development |
| F89 | Unspecified disorder of psychological development |
| F95 | Tic disorders |
| F99 | Mental disorder, not otherwise specified |
| **PARKINSON AND PARKINSONISM** | |
| **Included ICD-10 codes and labels** | |
| G20 | Parkinson disease |
| G21 | Secondary parkinsonism |
| G22 | Parkinsonism in diseases classified elsewhere |
| G23 | Other degenerative diseases of basal ganglia |
| **Excluded ICD-10 codes and labels** | |
| G210 | Malignant neuroleptic syndrome |
| **PERIPHERAL NEUROPATHY** | |
| **Included ICD-10 codes and labels** | |
| B91 | Sequelae of poliomyelitis |
| G14 | Postpolio syndrome |
| G54 | Nerve root and plexus disorders |
| G55 | Nerve root and plexus compressions in diseases classified elsewhere |
| G56 | Mononeuropathies of upper limb |
| G57 | Mononeuropathies of lower limb |
| G58 | Other mononeuropathies |
| G59 | Mononeuropathy in diseases classified elsewhere |
| G60 | Hereditary and idiopathic neuropathy |
| G628 | Other specified polyneuropathies |
| G629 | Polyneuropathy, unspecified |
| G63 | Polyneuropathy in diseases classified elsewhere |
| M472 | Other spondylosis with radiculopathy |
| M531 | Cervicobrachial syndrome |
| M541 | Radiculopathy |
| **Excluded ICD-10 codes and labels** | |
| G631 | Polyneuropathy in neoplastic disease |
| **PERIPHERAL VASCULAR DISEASE** | |
| **Included ICD-10 codes and labels** | |
| I702 | Atherosclerosis of arteries of extremities |
| I73 | Other peripheral vascular diseases |
| I792 | Peripheral angiopathy in diseases classified elsewhere |
| I798 | Other disorders of arteries, arterioles and capillaries in diseases classified elsewhere |
| **Excluded ICD-10 codes and labels** | |
| I731 | Thromboangiitis obliterans [Buerger] |
| I738 | Other specified peripheral vascular diseases |
| **SCHIZOPHRENIA AND DELUSIONAL DISEASES** | |
| **Included ICD-10 codes and labels** | |
| F20 | Schizophrenia |
| F22 | Persistent delusional disorders |
| F24 | Induced delusional disorder |
| F25 | Schizoaffective disorders |
| F28 | Other nonorganic psychotic disorders |

NOTE: When all sub-codes within a given ICD-10 code were classified as chronic, the highest possible level of aggregation of the hierarchy was included in the list (e.g. three-digit code for asthma (J45), one-digit code for malignant neoplasms (C), etc.).

Ad**ditional clinical and drug-related^*^ parameters used in SNAC-K for specific chronic conditions.**

| **Condition** | **Clinical and drug-related parameters** |
| --- | --- |
| **Atrial fibrillation** | Discrete P wave undetectable and irregular ventricular rate (12-lead electrocardiogram) |
| **Bradycardias and conduction diseases** | Presence of a cardiac pacemaker (12-lead electrocardiogram) |
| **Dementia** | Diagnostic and Statistical Manual of Mental Disorders, Third Edition, Revised^3^ (assessed by two different physicians, and a third one in case of disagreement)  Use of anticholinesterases (N06DA) or memantine (N06DX01) |
| **Ischemic heart disease** | Use of organic nitrates (C01DA) or ranolazine (C01EB18) |
| **Migraine and facial pain syndromes** | Use of antimigraine preparations (N02C) |
| **Other psychiatric and behavioral diseases** | Use of drugs for alcohol dependence (N07BB) |
| **Parkinson and parkinsonism** | Use of dopa and dopa derivatives (N04BA), dopamine agonists (N04BC), or other dopaminergic agents (N04BX) |
| **Peripheral vascular disease** | Use of cilostazol (B01AC23) |

^*^The ATC codes corresponding to each drug are shown in brackets. Only those drugs that can be unequivocally linked to chronic conditions were considered. That is, drugs with more than one indication were excluded from the list. The selection of ATC codes was based on a literature review and the clinical judgement of physicians.

NOTE: The criteria presented in this table were used in addition to the diagnoses assigned in SNAC-K. For example, use of dopaminergic agents was considered to indicate presence of Parkinson syndrome, even in the absence of other diagnostic information.
