# Supplementary material for: Trajectories of functional decline in older adults with neuropsychiatric and cardiovascular multimorbidity: A Swedish cohort study
Source: PLoS Med. 2018 Mar 6;15(3):e1002503. doi: 10.1371/journal.pmed.1002503 (PMC5839531; doi:10.1371/journal.pmed.1002503)
Supplement: S1 Study Protocol — (DOCX) [file pmed.1002503.s008.docx]

**S1 Study Protocol**

The present study is part of the four-year PhD project of the first author, which has been approved by the student’s supervisors and by the Committee for Doctoral Education of the Department of Neurobiology, Caring Sciences and Society of Karolinska Institutet, Sweden, at the beginning of the PhD program (January 7th 2016).

The title of the PhD project is “*Impact of cardiovascular and neuropsychiatric multimorbidity on older adults' health*”

Below we reported the sections of the PhD project more relevant for the present study. Compared with the original protocol, which included functional decline and mortality as outcomes, we later decided to focus our analyses on functional decline only, including walking speed (and not only disability) as outcome. Compared with the original plan we also decided to model our outcomes through linear-mixed models instead of Cox regressions, in order to get the most from the repeated measures of both walking speed and ADL.

“[…] GENERAL AND SPECIFIC AIMS

The overall goal of the present project is to better characterize the CV and NP multimorbidity, by exploring their occurrence, development, and prognosis in an elderly, community-based population. Our aims will be developed in four individual studies, to be completed in four years: […]

[…] STUDY III: Impact of cardiovascular and neuro-psychiatric multimorbidity on disability and mortality in older adults. The goal of this longitudinal study is to assess the effect of CV and NP multimorbidity on disability and mortality. We will specifically address the impact of these patterns of multimorbidity per se or in combination, comparing participants with and without CV and NP multimorbidity (two or more specific diseases). The interaction with other chronic diseases will be taken into account. Different measures of physical performance will be examined and related to the development of disability. […]

[…] STATISTICAL ANALYSES

Similarly, Cox proportional hazard model will be performed to examine the association between CV and NP multimorbidity with disability onset and mortality […]
